# Skin Permeation of Solutes from Metalworking Fluids to Build Prediction Models and Test A Partition Theory

**DOI:** 10.3390/molecules23123076

**Published:** 2018-11-24

**Authors:** Jacqueline M. Hughes-Oliver, Guangning Xu, Ronald E. Baynes

**Affiliations:** 1 Department of Statistics, North Carolina State University, Raleigh, NC 27695-8203, USA; hughesol@ncsu.edu; 2 Wells Fargo and Company, Charlotte, NC 28202-0901, USA; gxu@ncsu.edu; 3 Center for Chemical Toxicology Research & Pharmacokinetics, Department of Population Health and Pathobiology, College of Veterinary Medicine, North Carolina State University, 1060 William Moore Dr., Raleigh, NC 27607, USA

**Keywords:** leave-one-solute-out (LOSO) cross-validation, leave-one-out (LOO) cross-validation, linear free-energy relationship (LFER) model, membrane-coated fiber (MCF) approach, partition coefficient, quantitative structure-activity relationship (QSAR), metalworking fluid

## Abstract

Permeation of chemical solutes through skin can create major health issues. Using the membrane-coated fiber (MCF) as a solid phase membrane extraction (SPME) approach to simulate skin permeation, we obtained partition coefficients for 37 solutes under 90 treatment combinations that could broadly represent formulations that could be associated with occupational skin exposure. These formulations were designed to mimic fluids in the metalworking process, and they are defined in this manuscript using: one of mineral oil, polyethylene glycol-200, soluble oil, synthetic oil, or semi-synthetic oil; at a concentration of 0.05 or 0.5 or 5 percent; with solute concentration of 0.01, 0.05, 0.1, 0.5, 1, or 5 ppm. A single linear free-energy relationship (LFER) model was shown to be inadequate, but extensions that account for experimental conditions provide important improvements in estimating solute partitioning from selected formulations into the MCF. The benefit of the Expanded Nested-Solute-Concentration LFER model over the Expanded Crossed-Factors LFER model is only revealed through a careful leave-one-solute-out cross-validation that properly addresses the existence of replicates to avoid an overly optimistic view of predictive power. Finally, the partition theory that accompanies the MCF approach is thoroughly tested and found to not be supported under complex experimental settings that mimic occupational exposure in the metalworking industry.

## 1. Introduction

The assessment of skin permeation of chemical solutes can be used to inform scientific research and regulatory agencies in the risk management of chemical solutes that may be of concern especially for occupational exposures [1,2,3]. For example, in the metalworking industry, certain performance enhancing solutes such as corrosive inhibitors, emulsifiers, and biocides/preservatives are often added to the metalworking fluids (MWF). Contact with these industrial fluids containing some or all of these performance additives could sometimes cause skin irritation or even more harmful consequences [4,5,6,7]. Thus, it is of interest to study the permeation capability of the added solutes through skin, in the hopes of finding less permeable solutes that can be used in metalworking fluids.

Unfortunately, conducting skin absorption studies of the many industrial chemicals and many formulations can be very expensive, and many efforts have been made to mimic the skin using synthetic membranes [8,9,10,11,12,13]. Xia et al. [14] proposed an intriguing technique, called the membrane-coated fiber (MCF) assay approach, to simulate the different molecular interactions in skin permeation by different types of materials. In this approach, an MCF is used as the absorption membrane to determine partition coefficients, namely the ratio of the concentration of solute partitioning to the MCF relative to the concentration of solute not partitioning to the MCF. The partition coefficient is a measurement of the strength of molecular interaction that governs percutaneous absorption processes. Assuming that the MCF adequately represents skin absorption, larger values of partition coefficients suggest greater levels of absorption of the solute into skin, translating to possible health implications during the metalworking processes.

To relate the dermal permeability of a solute to the solute’s chemical structure or properties, it is very common practice to develop and study a relevant quantitative structure-activity relationship (QSAR) model as classically demonstrated by [15] and [16], and also demonstrated more recently in studies more relevant to this paper ([17,18,19]). Many commonly used QSAR models are linear regression models that use the biological activity (partition coefficients, permeation coefficients, etc.) as the response variable and the molecular descriptors as predictors. The linear free-energy relationship (LFER) model of [20] is a particular type of QSAR model that is widely used in modeling results from dermal permeability studies. The LFER model is easy to use and interpret, however, when experimental conditions are complex, a simple LFER model may not be able to appropriately account for the observed variability, leading to a model with poor fit statistics and low predictive power. Xu et al. [19] expanded the LFER model to account for the heterogeneity introduced by experimental factors, in which one set of partial slopes are defined for each experimental condition. This model proved to be useful, improving both the model fit statistics and predictive power. This article pursues extensions of the LFER model that are in the spirit of [19], but we are able to obtain further improvements in model performance by incorporating additional features observed in the current study. The critical role played by model assessment criterion$Q_{LOSO}^{2}$ is also reviewed. The resulting model provides interpretations that are useful for identifying solutes whose chemical structures are consistent with low predicted levels of skin permeability.

An attractive feature of the MCF approach of [14] is their proposed partition theory, namely that the partition coefficient of a solute from a formulation is not affected by the starting concentration of that solute in the formulation. This theory, if realized, can lead to simplified analysis even in the most complex of experimental conditions. By applying an expanded LFER model, we are able to test this theory that could not otherwise be tested.

Earlier efforts by Xia et al. 2007 [13] demonstrated the use of a MCF array to simulate skin permeability in simple binary mixtures. However the present paper utilizes the MCF and molecular structure parameters within an LFER model described above to now better estimate the effects of several real world formulations at various concentrations on the partitioning behavior of 37 solutes at different concentrations in an effort to estimate solute partitioning into MCF which serves as a surrogate for skin permeability

## 2. Results and Discussion

### 2.1. Data Summaries

Formulations are designed to mimic fluids used in the metalworking process. For this article, a *formulation* refers to: a particular metalworking fluid (MWF), at a particular MWF concentration, spiked with a solute at a particular concentration. Formulations are spiked with trace levels of solutes in such a way that the chemistry of the MWF is not altered.

In this study, we considered 37 solutes (see Table 1) and five solvatochromic descriptors believed to be most relevant to the solvation process during permeation [16,20]. These descriptors represent different characteristics of compounds involved in the solvation process, specified as follows. *E* is the solute excess molar refractivity, *S* is the solute dipolarity/polarizability, *A* is the overall hydrogen bond acidity, *B* is the overall hydrogen bond basicity, and *V* is the McGowan characteristic volume. For most solutes, *V* can be calculated directly, *E* can be obtained from experiment or calculated, but *A*, *B*, and *S* must be experimentally derived.

We varied the three other factors to create a formulation: the MWF, MWF concentration, and solute concentration. Five MWFs were considered: mineral oil (MO), polyethylene glycol-200 (PEG), soluble oil (SO), synthetic oil (SYN), and semi-synthetic oil (SSYN). MWF concentrations were at three levels: 0.05 percent, 0.5 percent, and 5 percent. Six solute concentrations were considered: 0.01, 0.05, 0.1, 0.5, 1, and 5 ppm. As a result, there were 5 × 3 × 6 = 90 treatment combinations, as displayed in Table A1 in Appendix A.

The study was designed to obtain partition coefficients, $K_{MCF/mix}$, for all 37 solutes, under each of the 90 treatment combinations, using three replicates. Unfortunately, due to a variety of reasons (e.g., lack of detection in gas chromatography, records outside the calibration range, etc.), not all replicates were recordable, with some treatment combinations even ending in no replicates for a particular solute. Fitting the QSAR model does not require replicates because of the structure provided by the model, and all collected data informs the fitting process. Having replicates would likely result in smaller measures of variability and hence greater power to make inference beyond what could done here, but the lack of replicates has not impeded the ability to conduct statistical analysis and model building. Of the maximum possible 37 × 90 × 3 = 9990 observations, we actually generated 4646 partition coefficients.

**Figure 1:**
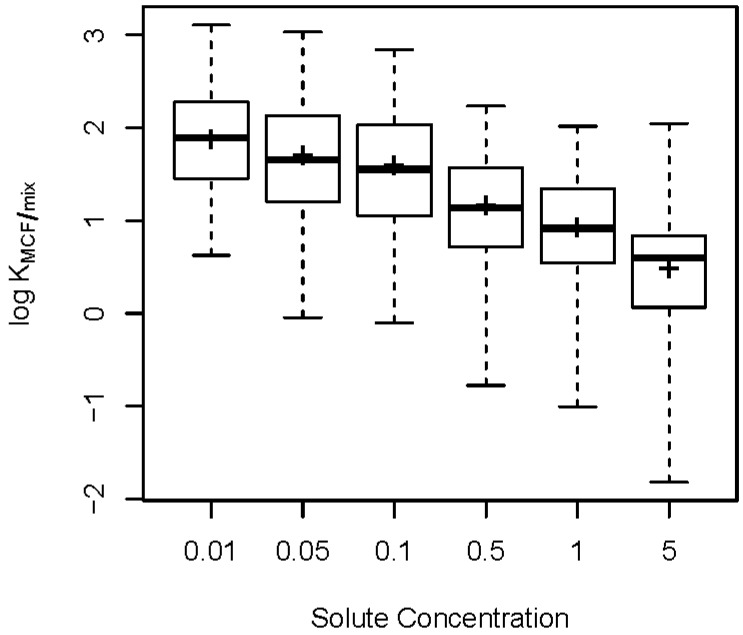
Boxplots of $\log\;K_{MCF/mix}$ across different solute concentrations. Thick horizontal lines are the medians, the means are shown as +, boxes contain the middle half of the data, and dotted lines extend to the minimum and maximum.

Summary statistics are displayed in Table 2 for all variables, based on the complete dataset of 4646 observations. Partition coefficients range from 0.015 to 1279 (−1.820 to 3.107 on the base 10 logarithm scale). To get a more detailed view of the range of values for partition coefficients, Figure 1 shows boxplots of $\log\;K_{MCF/mix}$ grouped by solute concentration. It is somewhat surprising that the smallest partition coefficients are associated with higher concentrations of solute present in the formulation; we return to this observation later in the article.

### 2.2. Insufficiency of the LFER Model

Abraham and Martins [20] proposed the general linear free-energy relationship (LFER) model to study dermal absorption:

$$SP=\beta_{0}+\beta_{1}E+\beta_{2}S+\beta_{3}A+\beta_{4}B+\beta_{5}V,$$

where *SP* is the property of interest for the solutes (such as $\log\;K_{p}$, log P, etc.). Given data, the coefficients in the LFER model are determined by multiple linear regression. These coefficients are also commonly denoted as *c*, *e*, *s*, *a*, *b*, and *v*; we used $\beta_{0}$, $\beta_{1}$, $\beta_{2}$, $\beta_{3}$, $\beta_{4}$, and $\beta_{5}$ as this is more common in the literature of multiple linear regression. In this article, logarithm of the partition coefficient, $\log\;K_{MCF/mix}$, is the property of interest. The resulting LFER model is shown in Equation (1):

(1) $$\log\;K_{MCF/mix}=\beta_{0}+\beta_{1}E+\beta_{2}S+\beta_{3}A+\beta_{4}B+\beta_{5}V.$$

While the LFER model in Equation (1) is simple and easy to interpret, it is not always sufficient, especially for large datasets under complicated experimental conditions. Equation (1) suggests that the expected value of $\log\;K_{MCF/mix}$ is a function of only *E*, *S*, *A*, *B*, and *V*. However, as is clearly demonstrated in Figure 1, $\log\;K_{MCF/mix}$ decreases as solute concentration increases, suggesting that solute concentration should likely be included as a predictor in Equation (1); we return to this observation below.

Focusing for the moment on the LFER model, Equation (1) was separately applied to data from each of the 90 treatment combinations, resulting in 90 separate estimated models. If all 90 estimated models essentially coincide, then the LFER model that only accounts for *E*, *S*, *A*, *B*, and *V*, and does not adjust for experimental conditions, is sufficient. To investigate this, Table 3 presents details on three of the 90 estimated models; details include estimated coefficients, their standard errors, and associated 95 percent confidence intervals. Estimated models are shown for: treatment combination 5, with mineral oil at 0.05 percent and solute concentration 1 ppm; treatment combination 17, with mineral oil at five percent and solute concentration 1 ppm; and treatment combination 52, with soluble oil at five percent and solute concentration 0.5 ppm.

**Figure 2:**
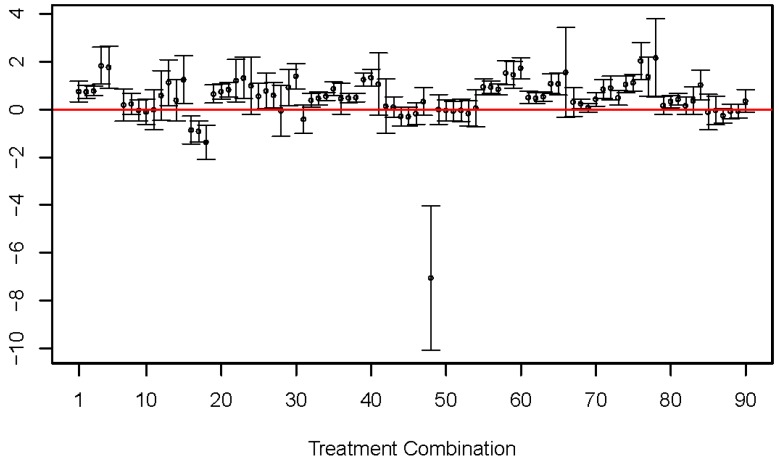
Estimated $\beta_{1}$ coefficients (circles) for molecular descriptor E from fitting separate LFER models across the 90 treatment combinations. Ninety-five percent (95%) confidence intervals are also shown, as vertical lines with two bars at the ends.

The estimated models in Table 3 did not coincide. Consider, for example, the coefficient $\beta_{1}$ corresponding to *E*. For treatment combination 5, the 95 percent confidence interval consists of only positive values (0.89 to 2.65), suggesting that $\log\;K_{MCF/mix}$ is expected to increase as excess molar refractivity increases. On the other hand, the 95 percent confidence interval consists of only negative values (−1.35 to −0.48) for treatment combination 17, suggesting that $\log\;K_{MCF/mix}$ is expected to decrease as excess molar refractivity increases. These conflicting interpretations are not isolated. Figure 2 graphs the 95 percent confidence intervals for coefficient $\beta_{1}$ corresponding to *E* from all 90 treatment combinations, and these intervals clearly do not coincide. Moreover, similar results hold for all coefficients, as demonstrated in Table 3.

### 2.3. Improvement by Expanded LFER Models

Xu et al. [19] demonstrate insufficiency of the LFER model for accounting for experimental conditions defined by four MWFs. They extend the LFER model by allowing for different sets of estimated coefficients for each of the four MWFs, all while using a single model. They obtained substantial improvements in predictive power of the Extended LFER model compared to the (single) LFER model. Hoping to achieve similar levels of improvement as [19], we also fitted an Extended LFER model that allows for different sets of estimated coefficients for each of the 90 treatment combinations, while using a single model, as follows:

$$\begin{array}{ll} \log\;K_{MCF/mix,ijkl} & =F_{1jkl}C_{i1kl}W_{ij1l}(\beta_{0111}+\beta_{1111}E_{l}+\beta_{2111}S_{l}+\beta_{3111}A_{l}+\beta_{4111}B_{l}\\ & +\beta_{5111}V_{l})\\ & +F_{1jkl}C_{i1kl}W_{ij2l}(\beta_{0112}+\beta_{1112}E_{l}+\beta_{2112}S_{l}+\beta_{3112}A_{l}+\beta_{4112}B_{l}\\ & +\beta_{5112}V_{l})+\cdots \\ & +F_{5jkl}C_{i3kl}W_{ij6l}(\beta_{0536}+\beta_{1536}E_{l}+\beta_{2536}S_{l}+\beta_{3536}A_{l}+\beta_{4536}\\ & +\beta_{5536}V_{l}), \end{array}$$

where $\log\;K_{MCF/mix,ijkl}$ is the *l*th observation from MWF *i* (*i =* 1 for MO, *i =* 2 for PEG, *i* = 3 for SO, *i* = 4 for SYN, and *i* = 5 for SSYN), MWF concentration *j* (*j =* 1 for 0.05, *j =* 2 for 0.5, and *j =* 3 for 5 percent), and solute concentration *k* (*k =* 1 for 0.01, *k =* 2 for 0.05, *k =* 3 for 0.1, *k =* 4 for 0.5, *k =* 5 for 1, and *k =* 6 for 5 ppm). In Equation (2), $\beta_{dijk}$ denotes the coefficient for descriptor *d* (with *d* = 0 for the intercept, *d =* 1 for *E*, *d =* 2 for *S*, *d =* 3 for *A*, *d =* 4 for *B*, and *d =* 5 for *V*) corresponding to MWF *i*, MWF concentration *j*, and solute concentration *k*. For example, $\beta_{1111}$ is the partial slope for descriptor *E* under treatment combination 1, with mineral oil at 0.05 percent and solute concentration 0.01 ppm. Three “dummy variables” $F_{ijkl}$, $C_{ijkl}$, and $W_{ijkl}$ are defined to indicate treatment combinations; these variables take value zero or one according to the levels of MWF, MWF concentration, and solute concentration. $F_{ijkl}=1$ if the observation comes from MWF *i*, otherwise $F_{ijkl}=0$; $C_{ijkl}=1$ if the observation comes from MWF concentration *j*, otherwise $C_{ijkl}=0$; and $W_{ijkl}=1$ if the observation comes from solute concentration *k*, otherwise $W_{ijkl}=0$.

The model in Equation (2) is quite large, having a maximum of 90 intercepts (one for each treatment combination) and 5 × 90 = 450 partial slopes (slopes corresponding to each of *E*, *S*, *A*, *B*, and *V* for each treatment combination). For any given observation, Equation (2) activates only a single set of coefficients because the product $F_{ijkl}C_{ijkl}W_{ijkl}$ will only be nonzero for a single treatment combination. For example, if the observation is in treatment combination 2 (mineral oil at concentration 0.05 percent with solute concentration 0.05 ppm), then $F_{1jkl}C_{i1kl}W_{ij2l}=1$ and all other $F_{ijkl}C_{ijkl}W_{ijkl}=0$, thus activating only $\beta_{0112}+\beta_{1112}E_{l}+\beta_{2112}S_{l}+\beta_{3112}A_{l}+\beta_{4112}B_{l}+\beta_{5112}V_{l}$ in Equation (2). Since Equation (2) is based on multiplying the dummy variables, we refer to it as the Expanded Crossed-Factors LFER model.

Table 4 shows regression statistics of fitting the Expanded Crossed-Factors LFER model of Equation (2). Regression statistics are also shown for the (single) LFER model of Equation (1), and another model to be described later. The improvements in r^2^, Adj-r^2^, $Q_{LOO}^{2}$, and $Q_{LOSO}^{2}$ are quite noticeable in favor of the Expanded Crossed-Factors LFER model over the LFER model. While r^2^ and Adj-r^2^ are widely known, $Q_{LOO}^{2}$, and $Q_{LOSO}^{2}$ may be less familiar. Both $Q_{LOO}^{2}$ and $Q_{LOSO}^{2}$ are designed to measure predictive ability of a model, but [19] demonstrate the advantage of $Q_{LOSO}^{2}$ over $Q_{LOO}^{2}$ for the current context. Leave-one-out (LOO) cross-validation is employed in both, meaning models are fit after reducing the dataset, then the resulting fit is used to make prediction on the portion of the data that was left out. The difference is that $Q_{LOSO}^{2}$ leaves out an entire solute at a time, whereas $Q_{LOO}^{2}$ omits a single row from the dataset. If only a single row is removed from the dataset, we are left with the possibility that a single replicate of a solute in a particular formulation may be removed, but the other two replicates remain in the dataset. The result is that the model is fit with almost full knowledge of the solute in question, and the consequence is that we are misled about the quality of the model for fitting “new, unseen” solutes. By removing every instance of a solute, $Q_{LOSO}^{2}$ provides a better assessment of the quality of the model for predicting new, unseen solutes. Large values are desirable for both $Q_{LOO}^{2}$ and $Q_{LOSO}^{2}$, but the extra demands placed on $Q_{LOSO}^{2}$ usually result in smaller values of $Q_{LOSO}^{2}$ compared to $Q_{LOO}^{2}$, in much the same way that Adj-r^2^ is often smaller than r^2^. (It is important to note that $Q_{LOSO}^{2}$ in this article is equivalent to $Q_{LOO-adj}^{2}$ in [19]. We prefer the simpler “*LOSO*” as it more clearly explains the difference from “*LOO*”.)

$Q_{LOO}^{2}$ is calculated as

(3) $$Q_{LOO}^{2}=1-\frac{\sum_{l=1}^{n}{\left(y_{l}-{\hat{y}}_{l,-l}\right)}^{2}}{\sum_{l=1}^{n}{\left(y_{l}-\bar{y}\right)}^{2}},$$

where $y_{l}$ is the *l*th observed response of $\log\;K_{MCF/mix}$, ${\hat{y}}_{l,-l}$ is the leave-one-out prediction of the *l*th observation based on the model fit without the *l*th observation, and $\bar{y\;}$ is the average of all the observed responses. $Q_{LOSO}^{2}$, designed by [19] to handle pseudo or real replicates in leave-one-out cross-validation for proper assessment of predictive power, is defined as:

(4) $$Q_{LOSO}^{2}=1-\frac{\sum_{s=1}^{37}\sum_{l=1}^{n_{s}}{\left(y_{sl}-{\hat{y}}_{sl,-s}\right)}^{2}}{\sum_{s=1}^{37}\sum_{l=1}^{n_{s}}{\left(y_{sl}-\bar{y}\right)}^{2}},$$

where $y_{sl}$ is the *l*th observation of the *s*th solute, $\bar{y}$ is the average of all the observed responses, and ${\hat{y}}_{sl,-s}$ is the predicted value of $y_{sl}$ based on the model fit from leaving out all the observations belonging to the *s*th solute.

While $Q_{LOSO}^{2}$ showed improvement of the Expanded Crossed-Factors LFER model over the LFER model, the value of 0.68 is not impressive and indicates some deficiency of the model. One possible reason may be overfitting. With so many regression para meters, this model seems to fit the data too closely, thus the idiosyncrasies of the data are captured instead of the general trends. The problem of overfitting is that when the model is applied to a new dataset, it cannot predict the new data well, as indicated by the weak value of $Q_{LOSO}^{2}$. This motivates us to look for an alternative model, which not only accounts for the heterogeneity introduced by different experimental conditions, but is also simpler and more predictive. The LFER model may be expanded in a variety of ways that accommodate experimental conditions, and the goal is to identify the simplest adequate expansion. As previously mentioned, the Expanded Crossed-Factors LFER model of Equation (2) is quite large, and we wondered whether it could be simplified.

**Figure 3:**
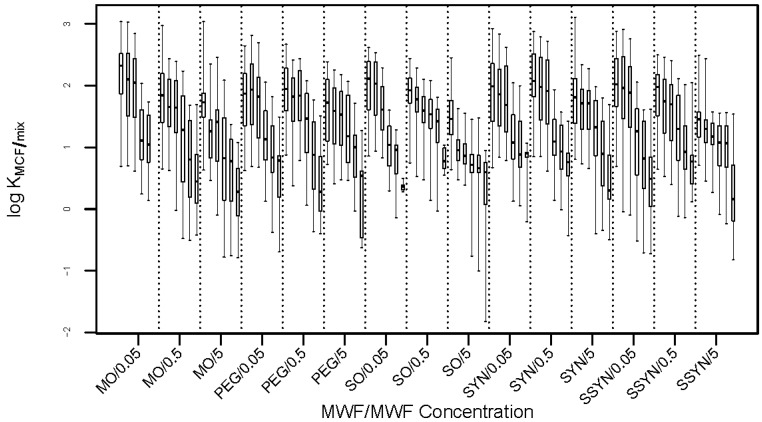
Boxplots of $\log\;K_{MCF/mix}$ across different solute concentrations in each of the 15 combinations of MWF and MWF concentration. Within each of the 15 panels, boxplots are shown for solute concentrations of 0.01, 0.05, 0.1, 0.5, 1, and 5 ppm. The 15 combinations (MWF/MWF concentration), from left to right, are: MO/0.05, MO/0.5, MO/5, PEG/0.05, PEG/0.5, PEG/5, SO/0.05, SO/0.5, SO/5, SYN/0.05, SYN/0.5, SYN/5, SSYN/0.05, SSYN/0.5, and SSYN/5.

Figure 1 tells us that partition coefficients decrease as the solute concentration increases. This suggests that there may be a quantifiable relationship between $\log\;K_{MCF/mix}$ and solute concentration. However, Figure 1 is the overall effect of solute concentration, not accounting for the effect of MWF or MWF concentration. Thus, a more detailed visualization is desired. Figure 3 depicts the trend of $\log\;K_{MCF/mix}$ over solute concentration in all 15 combinations of MWF and MWF concentration. It shows a similar trend as in Figure 1, for each of the 15 combinations of MWF and MWF concentration. Figure 3 suggests that instead of viewing solute concentration as a third factor crossed with MWF and MWF concentration, we can take it as a (numerically) nested factor within each of the combinations of MWF and MWF concentration. In other words, for each combination of MWF and MWF concentration, allow a different partial slope for solute concentration. By doing this, we place a structure within each MWF x MWF concentration condition, and may be able to see how $\log\;K_{MCF/mix}$ changes as a function of solute concentration.

We propose a new Expanded Nested-Solute-Concentration LFER model as in Equation (5):

(5) $$\begin{array}{ll} \log\;K_{MCF/mix,ijl} & =F_{1jl}C_{i1l}\left(\beta_{011}+\beta_{111}E_{l}+\beta_{211}S_{l}+\beta_{311}A_{l}+\beta_{411}B_{l}+\beta_{511}V_{l}+\beta_{611}t_{l}\right)\\ & +F_{1jl}C_{i2l}\left(\beta_{012}+\beta_{112}E_{l}+\beta_{212}S_{l}+\beta_{312}A_{l}+\beta_{412}B_{l}+\beta_{512}V_{l}+\beta_{612}t_{l}\right)+\cdots \\ & +F_{5jl}C_{i3l}\left(\beta_{053}+\beta_{153}E_{l}+\beta_{253}S_{l}+\beta_{353}A_{l}+\beta_{453}B_{l}+\beta_{553}V_{l}+\beta_{653}t_{l}\right), \end{array}$$

where $\log\;K_{MCF/mix,ijl}$ is the *l*th observation from MWF *i*, MWF concentration *j*, $t_{l}$ is the logarithm (base 10) of solute concentration of the *l*th observation, $\beta_{dij}$ is the regression coefficient of descriptor *d* (*d =* 0 for intercept, *d =* 1 for *E*, *d =* 2 for *S*, *d =* 3 for *A*, *d =* 4 for *B*, *d =* 5 for *V*, and *d =* 6 for logarithm of solute concentration), for MWF *i* and MWF concentration *j*. We take the logarithm of solute concentration as it is common practice and it linearizes the relationship. This model is relatively small, with a maximum of 15 × 7 = 105 coefficients to be estimated, compared to a maximum of 540 for the model in Equation (2).

Regression statistics are shown in Table 4, and it is clear that the Expanded Nested-Solute-Concentration LFER model of Equation (5) is at least as good as the Expanded Crossed-Factors LFER model of Equation (2), because it has comparable or larger values for all regression statistics. However, the Expanded Nested-Solute-Concentration LFER model of Equation (5) has a tremendous advantage in that: (1) it is much smaller, and so more amenable to interpretation; and (2) it is more predictive as indicated by a much larger value for $Q_{LOSO}^{2}$.

**Figure 4:**
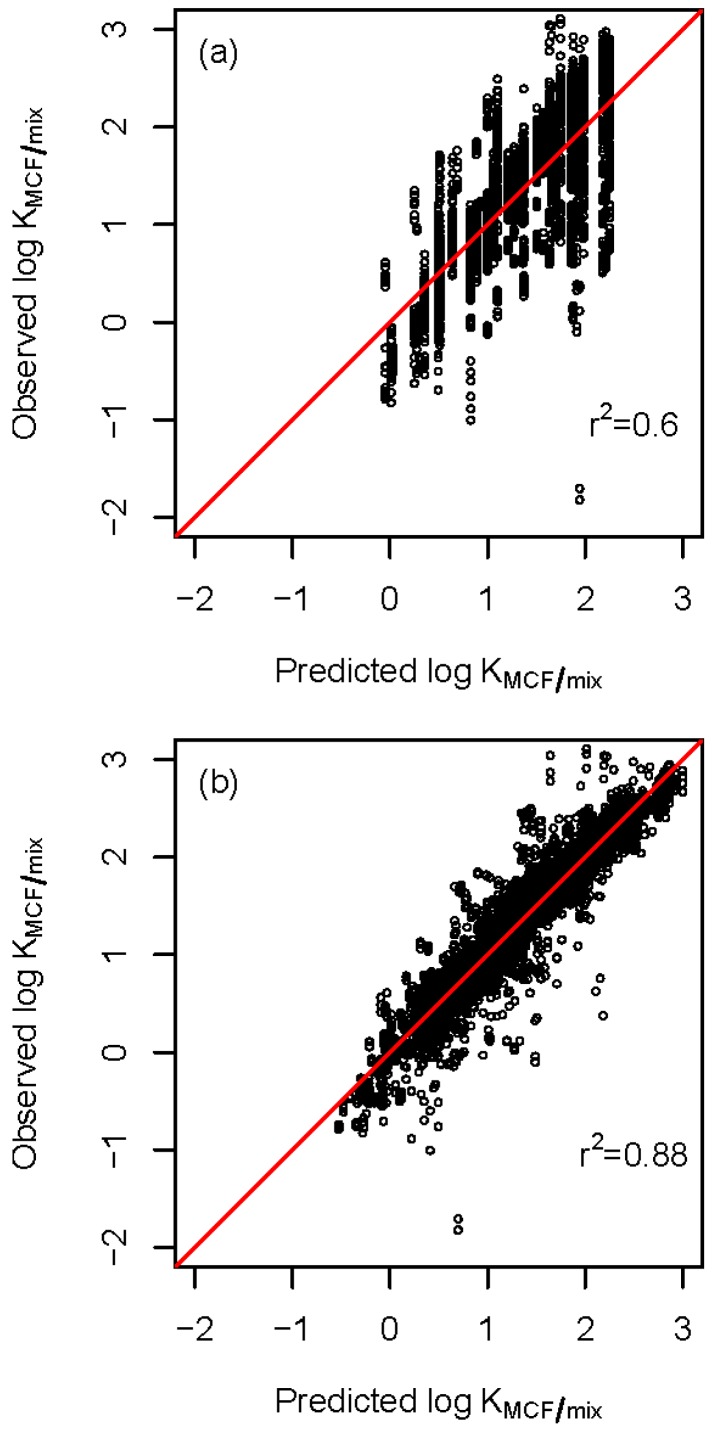
Observed versus predicted $\log\;K_{MCF/mix}$ for (**a**) the LFER model of Equation (1) and (**b**) the Expanded Nested-Solute-Concentration LFER model of Equation (5). Tightness around the line is indicative of a more predictive model.

Figure 4 plots observed versus predicted $\log\;K_{MCF/mix}$ values for both the LFER and Expanded Nested-Solute-Concentration LFER models. The tighter grouping around the line for the latter model is yet another demonstration of that model’s better predictive power.

### 2.4. Model Interpretation

We now intepret the estimated Expanded Nested-Solute-Concentration LFER model of Equation (5).

There are 15 rows in Equation (5), each representing the regression function for one combination of MWF/MWF concentration. For example, row one is for MWF mineral oil at concentration 0.05 percent, while row 15 is for MWF semi-synthetic oil at concentration five percent. Each row has a set of partial slopes that vary among the different combinations of MWF/MWF concentration. The estimates and associated standard errors of all partial slopes are shown in Table A2 in Appendix A.

**Figure 5:**
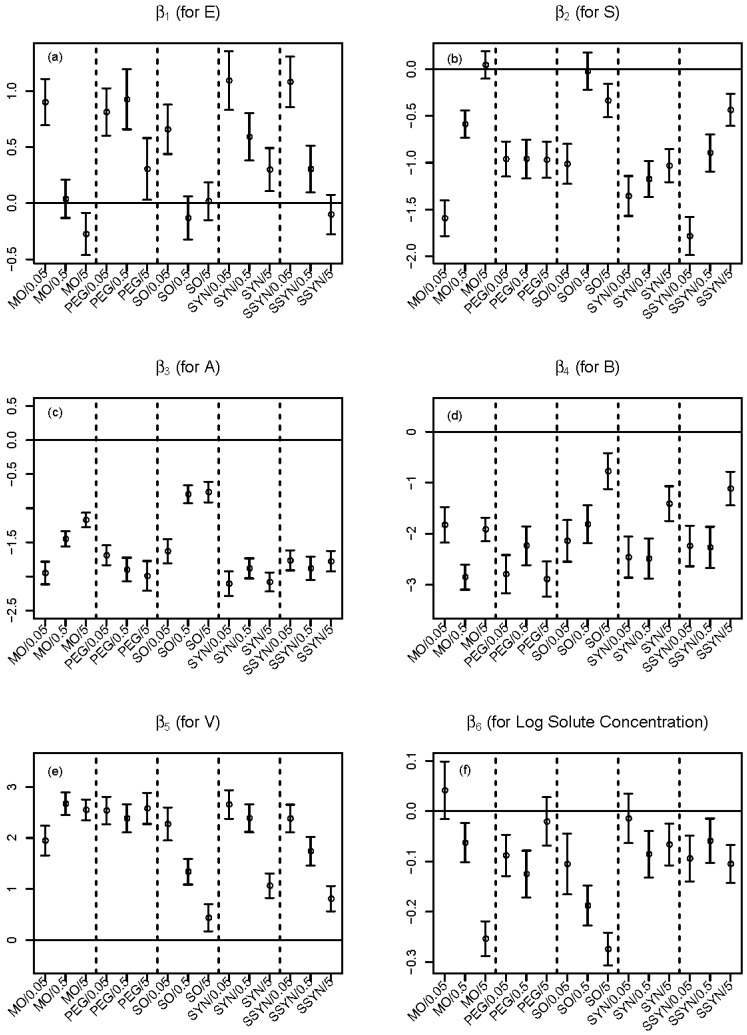
Estimated partial slopes (circles) corresponding to (**a**) *E*, (**b**) *S*, (**c**) *A*, (**d**) *B*, (**e**) *V*, and (**f**) log solute concentration from fitting the Expanded Nested-Solute-Concentration LFER model of Equation (5), for all 15 combinations of MWF/MWF concentration. Ninety-five percent (95%) confidence intervals are also shown, as vertical lines with two bars at the ends.

To show how the partial slopes vary, in Figure 5 we plot 95 percent confidence intervals for each partial slope corresponding to *E*, *S*, *A*, *B*, *V* and log solute concentration across all 15 combinations of MWF/MWF concentration. The 95 percent confidence intevals are shown as vertical lines with two bars at the ends. A horizontal reference line of zero is also shown. There are some interesting trends seen in Figure 5.

For example, in Figure 5a, the partial slope of *E* generally decreases as MWF concentration increases within each MWF. In mineral oil, the effect (sign of $\beta_{1}$) of *E* (solute excess molar refractivity) even changes as MWF concentration increases. To be specific, using mineral oil at concentration of 0.05 percent, if we increase solute excess molar refractivity and other predictors are held fixed, then the partition coefficient is expected to increase (the 95 percent confidence interval lays above the reference line). On the other hand, using mineral oil at the higher concentration of five percent, if we increase solute excess molar refractivity, then we expect the partition coefficient to decrease (the 95 percent confidence interval lays below the reference line).

In Figure 5b, the partial slope of *S* generally increases as MWF concentration increases within mineral oil, soluble oil, and semi-synthetic oil, but partial slopes show no significant change as MWF concentration increases within polyethylene glycol-200 and synthetic oil. In general, *S* (solute dipolarity/polarizability) has an inverse relationship with expected partition coefficient, meaning that as *S* increases we expected a decrease in partition coefficient.

Figure 5c suggests increased levels of hydrogen bond acidity *A* are associated with decreased partition coefficients. However, the pattern of decrease changes according to the concentration of MWF. For example, in both mineral oil and soluble oil, higher MWF concentrations result in smaller decrease in partition coefficients. Figure 5d indicates that increased levels of hydrogen bond basicity *B* generally leads to decreased partition coefficients.

Figure 5e says larger molecules tend to have larger partition coefficients. In soluble oil, synthetic oil and semi-synthetic oil, the effect of molecule size *V* gets smaller as MWF concentration increases, resulting in less dramatic effect of molecule size on partition coefficients.

Figure 5f suggests that higher concentrations of solute generally result in lower partition coefficients. In both mineral oil and soluble oil, higher MWF concentrations result in stronger inverse relationships.

### 2.5. Validation of Partition Theory

#### 2.5.1. Implication of Partition Theory

According to [14], it is assumed that the amount of solute extracted from the MCF, $n^{0}$, is proportional to the solute concentration, $C_{0}$, where the proportionality constant is not affected by $C_{0}$. Based on this assumption, we obtain $n^{0}=pC_{0}$, where *p* is the proportionality constant and 0≤p≤1. Applying this relationship to partition coefficients, we obtain:

(6) $$K_{MCF/mix}=\frac{n^{0}V_{d}}{V_{m}\left(C_{0}V_{d}-n^{0}\right)}=\frac{pC_{0}V_{d}}{V_{m}\left(C_{0}V_{d}-pC_{0}\right)}=\frac{pV_{d}}{V_{m}\left(V_{d}-p\right)}.$$

Equation (6) suggests that $K_{MCF/mix}$ is independent of $C_{0}$, which suggests that irrespective of the solute concentration, the partition coefficient remains the same. This so-called “partition theory”, if true, has practical meaning in the metalworking industry as it would indicate that increasing solute concentration has no impact on skin permeation ability of the solute. For example, higher concentrations of biocides might be preferred to extend preservation of fluids, while there is no detrimental effect of increasing the biocide’s ability to permeate skin. As described in more detail in the methods section, the MCF consists of a PDMS coating that is 100 µm thick and 1 cm long on an inert silica fiber. Solute partitioning into this membrane is dependent on the many chemical-chemical interactions quantified by our Expanded LFER models. However, the membrane volume (*V_m_*) suggests that this may be a limitation with increasing solute concentration. It was, therefore, interesting to see if this partition theory is supported by our data.

#### 2.5.2. Violation from Experimental Data

Assume the Expanded Nested-Solute-Concentration LFER model of Equation (5). To test whether the partition theory holds, we simply tested whether the coefficients corresponding to any solute concentration terms are different from zero. If all coefficients corresponding to solute concentration terms equal zero in Equation (5), then $\log\;K_{MCF/mix}$ will not change as solute concentration changes. More specifically, we test the following null hypothesis:

H_0_: *β*_6*ij*_ = 0 for all *i* = 1, 2, 3, 4, 5 and *j* = 1, 2, 3.

The resulting *p*-value of less than 0.0001 allows us to strongly conclude that the solute concentration term for at least one combination of MWF/MWF concentration is significantly different from zero. In fact, the individual P-values for testing each $\beta_{6ij}=0$ show that the solute concentration effect is significantly different from zero for 12 of the 15 combinations; nonsignificance is obtained only in MO/0.05, PEG/5 and SYN/0.05. These results are consistent with Figure 5f, where confidence intervals contain zero only for mineral oil at concentration 0.05, polyethylene glycol-200 at concentration 5 and synthetic oil at concentration 0.05.

Hoping to find that the partition theory holds true in either low or high solute concentrations, we considered subsets of data that contain only some of the solute concentrations. Detailed results are given in Table 5 of testing the null hypothesis that the partition theory holds for a number of different subsets of solute concentrations. For example, does the partition theory hold when considering only observations with solute concentrations less than or equal to 1 ppm? The answer is provided by row two of Table 5: with a *p*-value of less than 0.0001, the partition theory does not hold for solute concentrations less than or equal to 1 ppm, with violations happening in eight of the 15 combinations. In fact, the partition theory is violated in all subsets of solute concentrations.

## 3. Materials and Methods

**Figure 6:**
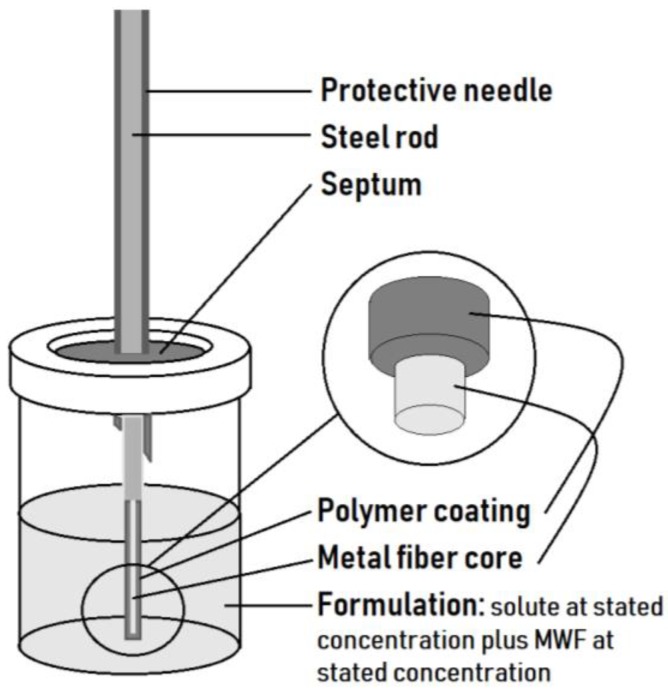
Membrane–coated fiber (MCF) and experimental setup. MWF = metal working fluid and the polymer coating is 100 µm thick polydimethylsiloxane (PDMS) that is part of the MCF.

Our experiments were based on the MCF approach proposed in [14]. Only a single MCF was used, namely PDMS (polydimethylsiloxane). In the current study, solutes were dissolved into a particular formulation, then an MCF was placed in the vial to allow the solute to partition from the solute-spiked formulation into the MCF over a period of one to four hours; see Figure 6. Gas chromatography and mass spectrometry were then used to extract or desorb the solute from the MCF, and the amount extracted was recorded.

### 3.1. Solvent/Solute Preparation

Three industry generic metal working fluids (MWF) formulations; soluble oil, synthetic fluid, and semi-synthetic fluid were kindly supplied by from Cimcool Industrial Products LLC (Cincinnati, OH, USA). The precise composition for each of these three formulations is proprietary information. In general, soluble oil concentrates contained approximately 58% mineral oil along with various other performance additives such as sulfonates and ethanolamines, semi-synthetic fluid concentrates contained about 15% mineral oil along with other additives such as sulfonates and ethanolamines, and synthetic fluid concentrates contain no mineral oil but contained various carboxylic acid salts, ethanolamines, ethyleneglycols, and plant seed oils. This is typical of many commercial MWF formulations that fall into these three categories. In addition to these three MWFs, two laboratory prepared surrogate formulations, mineral oil and PEG-200 (Aldrich, St. Louis, MO, USA) were prepared volumetrically in 0.05%, 0.5%, and 5.0% formulations in ultrapure water (Pure Water Solutions, Hillsborough, NC, USA). Each of these formulations were then spiked to six concentrations in the range of 0.01–5.0 µg/mL ranges with a set of 37 solutes (Table 1). These solutes were chosen to represent a wide variety of physiochemical properties. All solutes were of the highest purity available for purchase (Sigma Aldrich, Milwaukee, WI, USA). The 37 solutes were also prepared in acetone in a 2000 µg/mL stock solution. Experimental solutions were prepared fresh and all samples were kept at ambient temperature prior to analysis by SPME/GC-MS. Liquid GC-MS injections of the same 37 solutes prepared in acetone (0.01–10.00 µg/mL) were run daily, as well as blank liquid (acetone) and SPME (prepared solvent without addition of 37 solute) injections.

### 3.2. SPME/GC-MS Analysis

SPME absorption and injection was performed by a CTC Analytics Comi-Pal auto injector (Varian Inc., Walnut Creek, CA, USA) outfitted with a 100 µm polydimethylsiloxane SPME unit (Supelco Analytical, Bellafonte, PA, USA). A 9 mL sample was first agitated in a 37 °C heating block for 5 min, the SPME MCF (Figure 6) was then inserted and exposed for 30 min at 37 °C with constant agitation. SPME and liquid (0.5 µL) injections were introduced into a Varian 1079 injector (Varian Inc., Walnut Creek, CA, USA) at 280 °C in a split less mode for five min, at 5.5 min the split was turned on to 100%. For the first 30 seconds a pressure pulse of 21.0 psi was applied. Column flow was maintained at a constant 1.0 mL/min using helium as the carrier gas (National Welders, Raleigh, NC, USA). The Varian CP-3800 GC oven (Varian Inc., Walnut Creek, CA, USA) was programmed to hold at 40 °C for the first minute, followed by a 20 °C/min ramp to 90 °C (3.5 min), at which time the ramp slowed to 2.5 °C/min until 127 C (18.30 min) was reached and the ramp was increased to 40 C/min until it reached 250 °C and held for 2.0 min (23.38 min), followed by another increased ramp of 40 C/min until 280 °C and held for 5.0 min (29.13 min). The Saturn 2200-MS (Varian Inc., Walnut Creek, CA, USA) was programmed to run in full scan mode (40–300 *m*/*z*) after the first 3.0 min. Individual solute peaks were identified/quantified by the Star v6.5 software (Varian Inc., Walnut Creek, CA, USA) using retention time and known quant ions as identified and confirmed in the initial method development. Our sensitivity was set at 0.01 µg/mL as we were working with solutes ranging in concentrations from 0.01–5.0 µg/mL. More importantly, no residues were detected in the second injection after each first test injection, which indicated that there was negligible carry over under the optimum desorption conditions.

Differential ability of the solute to dissolve into the MCF or remain in the formulation was measured using a partition ratio (coefficient) $K_{MCF/mix}$ between the equilibrium concentration of the solute in the MCF and the equilibrium concentration of the solute in the formulation. $K_{MCF/mix}$ was calculated, following [14], as:

(7) $$K_{MCF/mix}=\frac{C_{pe}}{C_{me}}=\frac{n^{0}/V_{m}}{C_{0}-n^{0}/V_{d}}=\frac{n^{0}V_{d}}{V_{m}\left(C_{0}V_{d}-n^{0}\right)}$$

where $n^{0}$ is the amount (in μg) of solute extracted from the MCF, $V_{m}$ is the volume (in mL) of the MCF, $V_{d}$ is the volume (in mL) of formulation placed in the vial based on solute concentration $C_{0}$ (in μg/mL), $C_{pe}=n^{0}/V_{m}$ is the equilibrium concentration of solute in the MCF, and $C_{me}=C_{0}-n^{0}/V_{d}$ is the equilibrium concentration of solute in the formulation.

ADME Boxes 4.95, commercial software from ACD/Labs [21], was used to identify the *E*, *S*, *A*, *B*, and *V* descriptors for all the 37 solutes used in the experiment.

## 4. Summary and Conclusions

The partition theory of [14] does not appear to hold for the current study, as evidenced by Figure 1, Figure 3, and Table 5. It is probable that there is a finite number of binding sites available in the coating of the fiber (i.e., in the MCF). As the solute concentration increases, the percentage of the solute that absorbs and/or adsorbs to the membrane coating decreases due to this finite number of binding sites.

Notwithstanding the complications that arise from violations of the partition theory, our Expanded LFER models are able to adequately capture the variability of partition coefficients as a function of solute properties and experimental conditions. The Expanded Crossed-Factors LFER model based on [19] is a vast improvement over the single LFER model, while the Expanded Nested-Solute-Concentration LFER model developed in this article is even more refined, more predictive, and offers simple interpretations. Table 3, Table 4, Figure 2, and Figure 4 provide strong evidence that the simple LFER model is not adequate in the presence of complicated experimental conditions.

Proper assessment of model prediction ability is demonstrated with $Q_{LOSO}^{2}$ (previously $Q_{LOO-adj}^{2}$ in [19]), and this measure is contrasted with $Q_{LOO}^{2}$ and the more familiar r^2^ and Adj-r^2^. The leave-one-solute-out strategy allows assessment to occur based on completely unseen solutes.

## Figures and Tables

**Table 1:** Set of 37 solutes, and their descriptor values, used in this study.

Solute	Solute Name	*E*	*S*	*A*	*B*	*V*
1	Toluene	0.60	0.52	0	0.14	0.8573
2	Chloro-benzene	0.72	0.65	0	0.07	0.8388
3	Ethylbenzene	0.61	0.51	0	0.15	0.9982
4	p-Xylene	0.61	0.52	0	0.16	0.9982
5	Bromo-benzene	0.88	0.73	0	0.09	0.8914
6	Propyl-benzene	0.60	0.50	0	0.15	1.1391
7	1-Chloro-4-methyl-benzene	0.71	0.74	0	0.05	0.9797
8	Phenol	0.81	0.89	0.60	0.30	0.7751
9	Benzonitrile	0.74	1.11	0	0.33	0.8711
10	4-Fluoro-phenol	0.67	0.97	0.63	0.23	0.7927
11	Benzyl alcohol	0.80	0.87	0.39	0.56	0.9160
12	Iodo-benzene	1.19	0.82	0	0.12	0.9746
13	Phenyl ester acetic acid	0.66	1.13	0	0.54	1.0726
14	2-Chloro-acetophenone	1.02	1.59	0	0.41	1.1363
15	Phenol, 4-methyl-	0.82	0.87	0.57	0.31	0.9160
16	Nitro-Benzene	0.87	1.11	0	0.28	0.8906
17	Methyl ester benzoic acid	0.73	0.85	0	0.46	1.0726
18	1-chloro-4-methoxy-benzene	0.84	0.86	0	0.24	1.0384
19	Phenylethyl alcohol	0.81	0.86	0.31	0.65	1.0569
20	3-Methylbenzyl alcohol	0.82	0.90	0.39	0.59	1.0569
21	4-Ethyl-phenol	0.80	0.90	0.55	0.36	1.0569
22	3,5-Dimethyl-phenol	0.82	0.84	0.57	0.36	1.0569
23	Ethyl ester benzoic acid	0.69	0.85	0	0.46	1.2135
24	2-Methyl-methyl ester benzoic acid	0.77	0.87	0	0.43	1.2135
25	Naphthalene	1.34	0.92	0	0.20	1.0854
26	3-Chloro-phenol	0.91	1.06	0.69	0.15	0.8975
27	p-Chloroaniline	1.06	1.13	0.30	0.31	0.9386
28	1-methyl-4-nitro-benzene	0.87	1.11	0	0.28	1.0315
29	1-(4-Chlorophenyl)-ethanone	0.96	1.09	0	0.44	1.1363
30	3-Bromo-phenol	1.06	1.13	0.70	0.16	0.9501
31	4-Chloro-3-methyl-phenol	0.92	1.02	0.67	0.22	1.0384
32	1-Methyl-naphthalene	1.34	0.92	0	0.20	1.2263
33	Biphenyl	1.36	0.99	0	0.26	1.3242
34	Chloroxylenol	0.93	0.96	0.64	0.21	1.1793
35	4-(1,1-Dimethylpropyl)-phenol	0.79	0.80	0.50	0.44	1.4796
36	o-Hydroxybiphenyl	1.55	1.40	0.56	0.49	1.3829
37	Clorophene	1.53	1.42	0.67	0.47	1.6462

**Table 2:** Summary statistics for all variables, based on the complete dataset of 4646 observations.

Variable	Minimum	Lower Quartile	Mean	Median	Upper Quartile	Maximum	Std Dev
$\log\;K_{MCF/mix}$	−1.820	0.841	1.329	1.380	1.879	3.107	0.719
*E*	0.600	0.710	0.862	0.800	0.960	1.550	0.225
*S*	0.500	0.800	0.928	0.900	1.110	1.590	0.266
*A*	0.000	0.000	0.120	0.000	0.000	0.700	0.232
*B*	0.050	0.150	0.293	0.280	0.440	0.650	0.146
*V*	0.775	0.939	1.058	1.038	1.136	1.646	0.170

**Table 3:** Results from fitting separate LFER models (Equation (1)) for each of three treatment combinations (T).

T		$\beta_{0}$ (*Intercept*)	$\beta_{1}$ (for *E*)	$\beta_{2}$ (for *S*)	$\beta_{3}$ (for *A*)	$\beta_{4}$ (for *B*)	$\beta_{5}$ (for *V*)
5	est(se)	0.21(0.45)	1.77(0.43)	−1.59(0.27)	−1.87(0.18)	−0.50(0.42)	1.61(0.32)
	ci	(−0.71, 1.12)	(0.89, 2.65)	(−2.14, −1.03)	(−2.23, −1.51)	(−1.36, 0.36)	(0.97, 2.25)
17	est(se)	−0.61(0.27)	−0.91(0.22)	0.42(0.18)	−1.14(0.12)	−2.00(0.24)	2.47(0.25)
	ci	(−1.15, −0.07)	(−1.35, −0.48)	(0.07, 0.77)	(−1.37, −0.91)	(−2.48, −1.53)	(1.97, 2.97)
52	est(se)	1.50(0.31)	−0.03(0.23)	−0.36(0.25)	−0.42(0.20)	−0.98(0.48)	−0.14(0.34)
	ci	(0.89, 2.11)	(−0.50, 0.44)	(−0.87, 0.15)	(−0.83, −0.02)	(−1.94, −0.02)	(−0.81, 0.53)

For each of the intercept, *E*, *S*, *A*, *B*, and *V*, the table provides: the estimated coefficient (est), the associated standard error (se), and a 95 percent confidence interval (ci) for the coefficient. With large differences in estimated coefficients for different treatment combinations, these estimated models indicate a clear dependency on treatment combinations.

**Table 4:** Fit statistics of a single LFER model (1), the Expanded Crossed-Factors LFER model (2) and the Expanded Nested-Solute-Concentration LFER model (5).

Regression Statistics	LFER Model (1)	Expanded Crossed-Factors LFER Model (2)	Expanded Nested-Solute-Concentration LFER Model (5)
r^2^	0.60	0.90	0.88
Adj-r^2^	0.60	0.89	0.87
$Q_{LOO}^{2}$	0.60	0.87	0.87
$Q_{LOSO}^{2}$	0.57	0.68	0.80

**Table 5:** Testing the null hypothesis that the partition theory holds for a number of different subsets of solute concentrations.

Solute Concentrations	*p*-Value for H_0_	Insignificant Conditions	Significant Conditions
**All**	< 0.0001	MO/0.05(265), PEG/5(246), SYN/0.05(256), SSYN/0.5(297)	MO/0.5(379), MO/5(397), PEG/0.05(313), PEG/0.5(261), SO/0.05(247), SO/0.5(341), SO/5(368), SYN/0.5(300), SYN/5(321), SYN/0.05(298), SSYN/5(357)
**0.01, 0.05, 0.1, 0.5, 1**	< 0.0001	MO/0.05(265), MO/0.5(345), PEG/0.05(279), PEG/5(218), SYN/0.05(240), SYN/5(287), SSYN/0.5(278)	MO/5(347), PEG/0.5(239), SO/0.05(242), SO/0.5(327), SO/5(292), SYN/0.5(283), SSYN/0.05(278), SSYN/5(303)
**0.01, 0.05, 0.1, 0.5**	< 0.0001	MO/0.05(226), MO/0.5(285), PEG/0.05(236), PEG/0.5(205), PEG/5(182), SO/0.05(212), SYN/0.05(199), SYN/0.5(243), SYN/5(234), SSYN/0.05(238), SSYN/0.5(246)	MO/5(261), SO/0.5(271), SO/5(219), SSYN/5(229)
**0.01, 0.05, 0.1**	< 0.0001	MO/0.05(183), PEG/0.05(185), PEG/0.5(165), PEG/5(135), SO/0.05 (174), SYN/0.05(157), SYN/0.5(197), SYN/5(172), SSYN/0.05(191), SSYN/0.5(183)	MO/0.5(205), MO/5(176), SO/0.5(192), SO/5(145), SSYN/5(159)
**0.05, 0.1, 0.5**	< 0.0001	MO/0.05(173), MO/0.5(223),MO/5(208), PEG/0.05(184), PEG/0.5(155), PEG/5(147), SO/0.05(153), SYN/0.05(156), SYN/0.5(186), SYN/5(187), SSYN/0.05(180), SSYN/0.5(196), SSYN/5(187)	SO/0.5(141), SO/5(102)
**0.01, 0.05**	< 0.0001	MO/0.05(118), PEG/0.05(117), PEG/5(85), SO/0.05(127), SO/0.5(122), SYN/0.05(98), SYN/0.5(124), SYN/5(110), SSYN/0.05(123), SSYN/0.5(115), SSYN/5(99)	MO/0.5(129), MO/5(110), PEG/0.5(106), SO/5(91)
**0.05, 0.1**	< 0.0001	MO/0.05(130), MO/0.5(143), MO/5(123), PEG/0.5(115), PEG/5(100), SO/0.05(115), SO/5(102), SYN/0.05(114), SYN/0.5(140), SYN/5(125), SSYN/0.05(133), SSYN/0.5(133), SSYN/5(117)	PEG/0.05(133), SO/0.5(141)

The subset of solute concentrations is shown in the first column, with *p*-value given in the second column. MWF/MWF concentrations that support the partition theory (meaning their individual *p*-values are larger than 0.05/15, where division by 15 is to adjust for multiple testing) are shown in the third column (with sample sizes in parentheses). MWF/MWF concentrations that violate the partition theory are shown in the last column (with sample sizes in parentheses). The partition theory is violated in every subset, with the greatest support for the partition theory being achieved when limiting solute concentration to 0.05 or 0.1 or 0.5 ppm as the largest subset.

## Appendix A

This appendix contains tables that describe experimental results.

**Table A1:** Ninety treatment combinations with observation counts: MO for mineral oil, PEG-200 for polyethyleneglycol-200; SO for soluble oil; SYN for synthetic oil, and SSYN for semi-synthetic oil. Ideally, each treatment combination would include 37 × 3 = 111 observed partition coefficients, but missing data issues result in N observations, with N specified in the table.

Treatment Combination	MWF	MWF Conc. (%)	Solute Conc. (ppm)	N
**1**	MO	0.05	0.01	53
**2**	MO	0.05	0.05	65
**3**	MO	0.05	0.1	65
**4**	MO	0.05	0.5	43
**5**	MO	0.05	1	39
**6**	MO	0.05	5	0
**7**	MO	0.5	0.01	62
**8**	MO	0.5	0.05	67
**9**	MO	0.5	0.1	76
**10**	MO	0.5	0.5	80
**11**	MO	0.5	1	60
**12**	MO	0.5	5	34
**13**	MO	5	0.01	53
**14**	MO	5	0.05	57
**15**	MO	5	0.1	66
**16**	MO	5	0.5	85
**17**	MO	5	1	86
**18**	MO	5	5	50
**19**	PEG	0.05	0.01	52
**20**	PEG	0.05	0.05	65
**21**	PEG	0.05	0.1	68
**22**	PEG	0.05	0.5	51
**23**	PEG	0.05	1	43
**24**	PEG	0.05	5	34
**25**	PEG	0.5	0.01	50
**26**	PEG	0.5	0.05	56
**27**	PEG	0.5	0.1	59
**28**	PEG	0.5	0.5	40
**29**	PEG	0.5	1	34
**30**	PEG	0.5	5	22
**31**	PEG	5	0.01	35
**32**	PEG	5	0.05	50
**33**	PEG	5	0.1	50
**34**	PEG	5	0.5	47
**35**	PEG	5	1	36
**36**	PEG	5	5	28
**37**	SO	0.05	0.01	59
**38**	SO	0.05	0.05	68
**39**	SO	0.05	0.1	47
**40**	SO	0.05	0.5	38
**41**	SO	0.05	1	30
**42**	SO	0.05	5	5
**43**	SO	0.5	0.01	51
**44**	SO	0.5	0.05	71
**45**	SO	0.5	0.1	70
**46**	SO	0.5	0.5	79
**47**	SO	0.5	1	56
**48**	SO	0.5	5	14
**49**	SO	5	0.01	43
**50**	SO	5	0.05	48
**51**	SO	5	0.1	54
**52**	SO	5	0.5	74
**53**	SO	5	1	73
**54**	SO	5	5	76
**55**	SYN	0.05	0.01	43
**56**	SYN	0.05	0.05	55
**57**	SYN	0.05	0.1	59
**58**	SYN	0.05	0.5	42
**59**	SYN	0.05	1	41
**60**	SYN	0.05	5	16
**61**	SYN	0.5	0.01	57
**62**	SYN	0.5	0.05	67
**63**	SYN	0.5	0.1	73
**64**	SYN	0.5	0.5	46
**65**	SYN	0.5	1	40
**66**	SYN	0.5	5	17
**67**	SYN	5	0.01	47
**68**	SYN	5	0.05	63
**69**	SYN	5	0.1	62
**70**	SYN	5	0.5	62
**71**	SYN	5	1	53
**72**	SYN	5	5	34
**73**	SSYN	0.05	0.01	58
**74**	SSYN	0.05	0.05	65
**75**	SSYN	0.05	0.1	68
**76**	SSYN	0.05	0.5	47
**77**	SSYN	0.05	1	40
**78**	SSYN	0.05	5	20
**79**	SSYN	0.5	0.01	50
**80**	SSYN	0.5	0.05	65
**81**	SSYN	0.5	0.1	68
**82**	SSYN	0.5	0.5	63
**83**	SSYN	0.5	1	32
**84**	SSYN	0.5	5	19
**85**	SSYN	5	0.01	42
**86**	SSYN	5	0.05	57
**87**	SSYN	5	0.1	60
**88**	SSYN	5	0.5	70
**89**	SSYN	5	1	74
**90**	SSYN	5	5	54

**Table A2:** Estimation details for the Expanded Nested-Solute-Concentration LFER model of Equation (5). Estimated coefficients (with standard errors in parentheses) are given that correspond to the 15 combinations of MWF/MWF concentrations of the nested model. The logarithm of solute concentration is denoted as *t*.

	$\beta_{0ij}$ (*Intercept*)	$\beta_{1ij}$ (for *E*)	$\beta_{2ij}$ (for *S*)	$\beta_{3ij}$ (for *A*)	$\beta_{4ij}$ (for *B*)	$\beta_{5ij}$ (for *V*)	$\beta_{6ij}$ (for *t*)
**MO/0.05**	1.15(0.15)	0.9(0.11)	−1.59(0.10)	−1.95(0.08)	−1.83(0.18)	1.95(0.15)	0.04(0.03)
**MO/0.5**	0.10(0.11)	0.04(0.09)	−0.59(0.07)	−1.45(0.06)	−2.85(0.13)	2.67(0.11)	−0.06(0.02)
**MO/5**	−0.86(0.10)	−0.27(0.1)	0.04(0.07)	−1.17(0.05)	−1.91(0.12)	2.55(0.10)	−0.25(0.02)
**PEG/0.05**	−0.12(0.11)	0.81(0.11)	−0.96(0.10)	−1.69(0.07)	−2.79(0.19)	2.54(0.14)	−0.09(0.02)
**PEG/0.5**	−0.10(0.11)	0.93(0.14)	−0.96(0.11)	−1.90(0.09)	−2.23(0.19)	2.39(0.14)	−0.13(0.02)
**PEG/5**	0.15(0.15)	0.3(0.14)	−0.97(0.10)	−1.99(0.11)	−2.88(0.18)	2.58(0.16)	−0.02(0.02)
**SO/0.05**	0.30(0.13)	0.66(0.11)	−1.01(0.11)	−1.63(0.09)	−2.14(0.21)	2.27(0.17)	−0.11(0.03)
**SO/0.5**	0.69(0.11)	−0.13(0.10)	−0.02(0.10)	−0.79(0.07)	−1.82(0.19)	1.34(0.13)	−0.19(0.02)
**SO/5**	0.74(0.12)	0.02(0.09)	−0.34(0.09)	−0.76(0.08)	−0.77(0.18)	0.44(0.14)	−0.27(0.02)
**SYN/0.05**	0.03(0.12)	1.09(0.13)	−1.36(0.11)	−2.11(0.09)	−2.46(0.20)	2.66(0.14)	−0.01(0.03)
**SYN/0.5**	0.53(0.11)	0.59(0.11)	−1.17(0.10)	−1.88(0.07)	−2.48(0.20)	2.39(0.14)	−0.09(0.02)
**SYN/5**	1.55(0.11)	0.30(0.10)	−1.03(0.09)	−2.08(0.07)	−1.41(0.17)	1.06(0.12)	−0.07(0.02)
**SSYN/0.05**	0.53(0.11)	1.08(0.11)	−1.78(0.10)	−1.77(0.07)	−2.24(0.20)	2.38(0.14)	−0.09(0.02)
**SSYN/0.5**	0.90(0.12)	0.30(0.11)	−0.90(0.10)	−1.88(0.09)	−2.27(0.20)	1.74(0.14)	−0.06(0.02)
**SSYN/5**	1.03(0.11)	−0.10(0.09)	−0.44(0.09)	−1.78(0.08)	−1.11(0.17)	0.81(0.13)	−0.10(0.02)
